# Development of an E2 ELISA Methodology to Assess Chikungunya Seroprevalence in Patients from an Endemic Region of Mexico

**DOI:** 10.3390/v11050407

**Published:** 2019-05-01

**Authors:** Young Chan Kim, César López-Camacho, Nallely Garcia-Larragoiti, Alan Cano-Mendez, Karina Guadalupe Hernandez-Flores, Carlos Alonso Domínguez-Alemán, Maria Antonieta Mar, Héctor Vivanco-Cid, Martha Eva Viveros-Sandoval, Arturo Reyes-Sandoval

**Affiliations:** 1 The Jenner Institute, Nuffield Department of Medicine, University of Oxford, The Henry Wellcome Building for Molecular Physiology, Roosevelt Drive, Oxford OX3 7DQ, UK; young.kim@some.ox.ac.uk (Y.C.K.); cesar.lopez-camacho@ndm.ox.ac.uk (C.L.-C.); 2 Division of Structural Biology, University of Oxford, Wellcome Centre for Human Genetics, Roosevelt Drive, Oxford OX3 7BN, UK; 3 Laboratorio de Hemostasia y Biología Vascular, División de Estudios de Posgrado, Facultad de Ciencias Médicas y Biológicas “Dr. Ignacio Chávez”, Universidad Michoacana de San Nicolás de Hidalgo, UMSNH, Morelia 58000, Mexico; garcia_larragoiti@hotmail.com (N.G.-L.); bqdalan@hotmail.com (A.C.-M.); marthaevaviveros@yahoo.com.mx (M.E.V.-S.); 4 UMSNH–Oxford University of Oxford Clinical Research Laboratory (UMOCRL), Faculty of Biological and Medical Sciences “Dr. Ignacio Chávez”, Universidad Michoacana de San Nicolás de Hidalgo, Morelia 58000, Mexico; 5 Instituto de Investigaciones Médico-Biológicas, Universidad Veracruzana, Región Veracruz, Veracruz 91700, Mexico; karinahernandezflores@gmail.com (K.G.H.-F.); aleman_815@hotmail.com (C.A.D.-A.); hvivanco@uv.mx (H.V.-C.); 6 Programa de Maestría en Ciencias de la Salud, Instituto de Ciencias de la Salud, Universidad Veracruzana, Xalapa, Veracruz 91190, Mexico; 7 Hospital General de Zona y Medicina Familiar (HGZMF) No. 12, Av. Lázaro Cárdenas No. 154 Col. Centro, Lázaro Cárdenas 60950, Mexico; maria.mar@imss.gob.mx

**Keywords:** Chikungunya virus, envelope protein 2, diagnosis, ELISA, febrile patients, CHIKV antibodies, Mexico

## Abstract

Chikungunya fever is a debilitating disease caused by Chikungunya virus (CHIKV) that can result in long-lasting arthralgias. The early diagnosis of CHIKV relies on PCR during the acute infection phase to allow differential diagnosis with other co-circulating arboviruses such as dengue and Zika. Alternatively, serology can support diagnosis and provide epidemiological information on current and past outbreaks. Many commercial serological ELISA assays are based on the inactivated whole CHIKV, but their sensitivity and specificity show great variability. We produced recombinant CHIKV E2 that is suitable for ELISA assays, which was used for the serodiagnosis of CHIKV infections occurring in an arbovirus endemic Mexican region within Michoacán state. A cross-sectional study was conducted in 2016–2017; sera was obtained from 15 healthy donors and 68 patients presenting undifferentiated febrile illness. Serum samples were screened by RT-PCR and by our in-house ELISA assay. Our results indicate that IgM and IgG anti-CHIKV E2 antibodies were detected with our ELISA assay with higher sensitivity than a commercially available CHIKV ELISA kit. Our simple and sensitive ELISA assay for the serodiagnosis of CHIKV infections can be applied to population-based seroprevalence surveys and has potential for monitoring vaccine immunogenicity in CHIKV vaccine clinical trials.

## 1. Introduction

Chikungunya virus (CHIKV) is a re-emerging arbovirus that belongs to the *Alphavirus* genus of the *Togaviridae* family [1,2,3]. CHIKV is transmitted to humans by *Aedes* spp. mosquitoes [3,4,5]. The genome of CHIKV consists of a positive-sense, single-stranded RNA of approximately 11.8 kb, and contains structural genes that encode three structural proteins: envelope proteins 1 and 2 (E1 and E2) and a nucleocapsid protein along with two small peptides (E3 and 6K) [6,7,8]. The CHIKV E1 and E2 form spikes composed of triplets of E1–E2 heterodimers covering the viral surface [9,10]. Since its appearance in 2013 in the Caribbean, it has rapidly spread throughout the Americas, raising public health concerns [11,12,13]. CHIKV infections are characterized by an acute onset of fever associated with myalgia, headache, and severe debilitating arthropathy [3]. Vaccines or specific anti-viral therapies for CHIKV are not yet available; thus, rapid and simple methodologies to detect CHIKV infections are important for effective patient management and the control of future epidemics [12,14]. The primary laboratory test to diagnose CHIKV infection relies on the specific detection of CHIKV genomic sequences in serum collected up to five to six days after the onset of fever [15,16,17]. However, serological tests are required for the confirmation of CHIKV infection after the end of the viremia. Several commercial and in-house CHIKV serological assays that became available are based on whole virus antigens, and those reports indicate a variation of sensitivities and specificities [18,19,20,21,22].

Anti-CHIKV IgM antibodies appear as early as three days after infection, but their presence is usually detected only after three to four months [18,23]. In contrast, anti-CHIKV IgG antibodies remain detectable in convalescent individuals for many years; thus, anti-CHIKV IgG are able to provide insights into the seroepidemiology of CHIKV [20,24]. However, it is often difficult to differentiate acute from convalescent cases based on the detection of specific IgG alone in highly endemic areas. Despite this difficulty, a recent multi-country quality assurance study showed that the IgG detection of CHIKV infection may be more useful than IgM detection [25]. Therefore, we sought to develop an improved diagnostic tool to detect CHIKV-specific IgM and IgG based on a recombinant subunit protein that may be important in improving the sensitivity of serodiagnosis for clinical management and epidemiological surveillance. We have recently reported a recombinant CHIKV E2 protein in mammalian cells [26]. Based on this, we sought to detect and quantify anti-CHIKV E2 IgM and IgG E2 in human sera from CHIKV acute infections or convalescent patients. In order to validate our in-house ELISA, we have used a cohort of sera from infected patients tested by a reverse transcription-polymerase chain reaction (RT-PCR) along with the sera from healthy volunteers, and then compared these to a commercial ELISA assay. Results showed that our in-house E2 based IgM and IgG ELISA assays could detect anti-CHIKV IgM and IgG antibodies with similar or higher sensitivity than the commercial kit. The data presented here also provide valuable serological information regarding the current seroprevalence of CHIKV in the city of Lázaro Cárdenas, state of Michoacán, in México.

## 2. Materials and Methods

### 2.1. Study Design

A descriptive cross-sectional study was conducted in October 2016 and June–August 2017 at outpatient clinics at Lázaro Cárdenas, a port city in the state of Michoacán, México, where arboviruses are endemic. A total of 68 samples were collected and processed for anti-CHIKV IgM and IgG ELISA. Fifteen samples from healthy donors were collected in Morelia, the capital city in the state of Michoacán, where the circulation of CHIKV is not considered to be endemic.

### 2.2. Sample Collection

Sera were obtained from febrile patients with signs or symptoms compatible with Chikungunya fever (fever, joint pain, or rash) in outpatient clinics at Lázaro Cárdenas at two different time points (seven samples in October 2016 and 61 samples in June–August 2017). These samples were tested with CHIKV RT-PCR to confirm the CHIKV infection. All the serum samples were initially kept at 4 °C, and after transportation to the laboratory, they were stored at −80 °C until further processing.

### 2.3. Real-Time Quantitative Reverse Transcription PCR (qRT-PCR)

Briefly, the extraction of viral RNA from serum samples was performed using a QIAamp Viral RNA Mini Kit (Qiagen, Germany) following the manufacturer’s instructions. A commercially available rtRT-PCR primer/probe kit (Path-CHIK-standard kits, Genesig, Primer design Ltd., Cambridge, UK) was used to detect and amplify Chikungunya RNA. All the serum samples were tested for Chikungunya RNA. The tests were performed using an Applied Biosystem step one real-time PCR machine (Applied Biosystem, CA, USA). A reaction mix with a final volume of 20 μL was prepared adding 10 μL of oasig One step PCR Master mix, 1 μL of CHIK primers/probe, 1 μL of internal extraction control primer/probe mix, 5 μL of viral RNA, and 3 μL of RNAse/DNAse free water. The cycling conditions for the RT-PCR consisted of a reverse transcription step of 55° for 30 min; enzyme activation for the PCR was performed at 95 °C for 2 min, followed by 50 cycles of denaturation 95 °C for 10 s, and data collection 60 °C for 60 s, as described by the manufacturer instructions. Fluorogenic data was collected through the FAM and VIC channels.

### 2.4. Production and Purification of Recombinant CHIKV E2 Protein

The production and purification of recombinant CHIKV E2 protein was carried out as described previously [26]. Briefly, the expression plasmid (500 μg) was individually transfected in HEK-293T cells using polyethyleneimine (PEI) in roller bottles (surface area of 2125 cm^2^) under standard cell culture conditions. Five days after transfection, cells were discarded and media was filtered through 0.22-μM disposable filters. The secreted proteins were purified from the supernatant by Niquel Sepharose affinity chromatography (HisTRAP^TM^, GE Healthcare Life Sciences, UK), using the Äkta Start chromatography system and eluted with 500 mM of imidazole. Finally, eluted proteins were dialyzed using a Slide-A-LyzerTM cassette (Fisher Scientific, UK) against 1× PBS.

### 2.5. Detection of Anti-CHIKV IgM and IgG by in-House ELISA

A total of 68 serum samples from febrile Mexican patients in an endemic area and 15 sera samples from healthy blood donors from a non-endemic area were tested in parallel. For the initial method development, seven patient sera from RT-PCR confirmed CHIKV infections were used for recombinant CHIKV E2-based IgG and IgM ELISA assay to measure the antibodies against E2 along with seven negative control sera from healthy volunteers. A CHIKV-positive control serum sample was obtained from a Mexican blood donor who was known to have confirmed CHIKV infection independent from this study. Briefly, human sera were diluted in Nunc Maxisorp Immuno ELISA plates coated with CHIKV E2 in PBS to a final concentration of 2 μg/mL. Following blocking, sera was incubated for 1 h; then, plates were washed six times with PBS/T (0.05%), and bound antibodies were detected by using a goat Anti-Human IgG–alkaline phosphatase-conjugated antibody (Sigma-Aldrich UK, A3187-.5ML) or a goat Anti-Human IgM (μ-chain specific)–alkaline phosphatase-conjugated antibody (Sigma, A3275-.5ML). Development was performed using 4-nitrophenylphosphate diluted in diethanolamine buffer, and absorbance values at OD405 were measured on a CLARIOstar instrument (BMG Labtech UK). Serum antibody endpoint titres for positive samples were defined by an absorbance value that was three standard deviations greater than the average OD405 of the negative controls.

### 2.6. Detection of Anti-CHIKV IgM and IgG by Commercial ELISA Kit

For the qualitative measurement of IgM and IgG class antibodies against CHIKV in human serum, an Anti-Chikungunya Virus IgM Human ELISA Kit (ab 177848) and an IgG Human ELISA Kit (ab 177835) from Abcam were used to test all the serum samples according to the manufacturer’s protocols. The results were calculated and interpreted as suggested in the protocol.

### 2.7. Maps and Geographical Data

**Figure 1:**
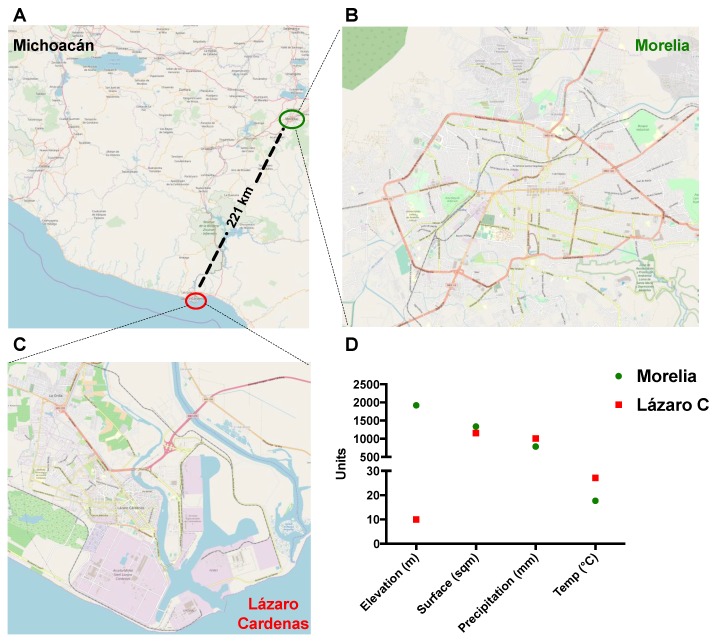
The geographical location of study sites. (**A**–**C**). Map of Michoacan State in Mexico showing the locations of study sites (Lazaro Cardenas and Morelia) and illustration of the geographical differences between the two municipalities. (**D**). Graph showing the elevation, surface, precipitation, and temperature between the two municipalities.

Maps in Figure 1A–C were obtained using the QGIS 3.6 software Release 3.6 and the QGIS Geographic Information System, using the ESRI satellite database. Then, maps were layered with the OpenStretMap option. Geographical and climate conditions were obtained from historical databases available at the Mexican system for geographical information and the National meteorological service websites, respectively.

### 2.8. Ethics Approval and Consent to Participate

The human sera statement was approved by the appropriate ethical review sub-committees, and written informed consent was obtained from all the study participants in Mexico (R-2016-785-104).

### 2.9. Data Analysis and Statistics

All the data analysis and statistics were performed using the Prism 7 software (GraphPad, Software, U.S.). *p* Values and R^2^ values reflect Pearson correlation tests for determining the correlation between the endpoint reciprocal titer values from ELISA assay and mean standard units (SU) from commercial kits.

## 3. Results

**Figure 2:**
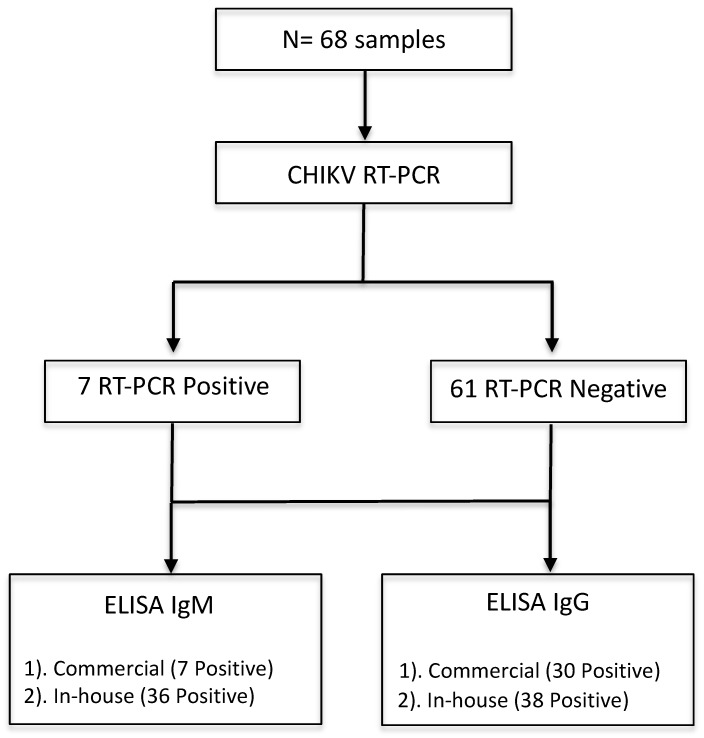
Flow chart describing tests performed in samples using RT-PCR and two ELISA methodologies.

During October 2016 and June–August 2017, 68 febrile patients were recruited in this study from Lázaro Cárdenas, Michoacán, México (Figure 1A). Lázaro Cárdenas is a port city and an arbovirus endemic region (Figure 1C) with low altitude and higher temperature than Morelia (Figure 1B,D). By following the algorithm depicted in Figure 2, we performed qRT-PCR to all 68 samples. Seven out of 68 samples (10.3%) tested positive on RT-PCR confirming the acute CHIKV infection.

### 3.1. Development and Evaluation of CHIKV Recombinant E2 Based in-House ELISA

**Figure 3:**
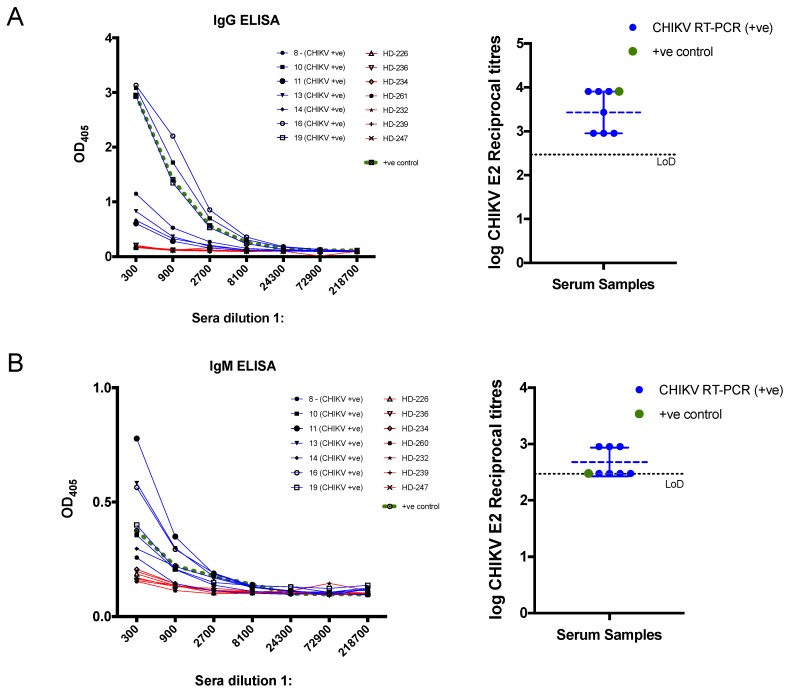
ELISA assays to assess the reactivity of human sera. Chikungunya virus (CHIKV)-infected patients (blue) confirmed by RT-PCR, sera obtained from healthy donors (HD) from a non-endemic region (red) and a CHIKV-positive control serum (green). (**A**) The graph of CHIKV E2 IgG OD405 against sera dilutions (left) and the corresponding endpoint log reciprocal titres (right). (**B**) The graph of CHIKV E2 IgM OD405 (left) and the corresponding endpoint log reciprocal titres (right). The black dotted line indicates the limit of detection (LoD), which corresponds to 1:300 serum dilution on a log scale.

Next, we sought to test whether our recombinant CHIKV E2 could be used to detect anti- CHIKV E2 antibodies in human serum. We selected the seven CHIKV RT-PCR positive samples (blue), a positive CHIKV control (green), and seven control sera (red) from healthy volunteers to test the suitability of our recombinant CHIKV E2 proteins in IgG and IgM-based ELISA assay (Figure 3). The OD405 demonstrate that both IgG and IgM-based ELISA can reliably detect and differentiate the sera from CHIKV-infected patients from control sera (Figure 3A left and Figure 3B left), but OD405 values were higher for IgG-based ELISA than for IgM. Endpoint reciprocal titres were calculated and showed that all confirmed CHIKV patients have high mean titres of 3.4 and 2.7 on IgG and IgM ELISA, respectively (Figure 3A right and Figure 3B right). These results indicate that our purified CHIKV E2 recombinant protein can reliably detect anti-CHIKV E2 antibodies in infected patients, which may be relevant for the serodiagnosis of CHIKV infection.

### 3.2. CHIKV Serological Diagnosis Using the Commercial ELISA Kits

**Figure 4:**
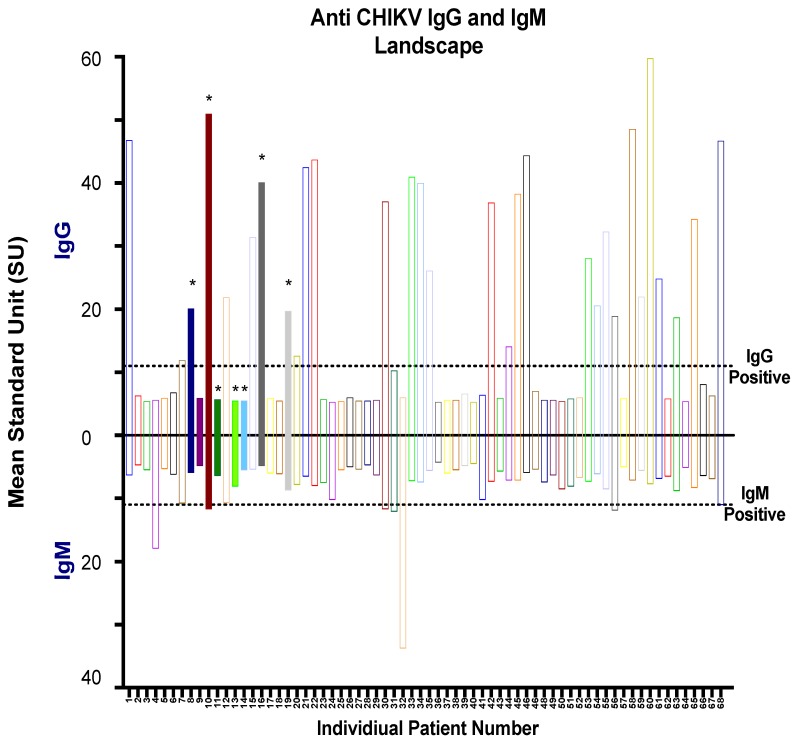
Qualitative ELISA assays using the commercial kits to assess the reactivity of human serum samples. Mean standard unit (SU) for IgG (positive *y*-axis) and IgM (negative *y*-axis) are represented for all 68 serum samples. Positive samples are defined as >11 SU, and the negative samples are defined as <9. The SU of samples 9–11 are defined as in the grey zone (inconclusive results). RT-PCR positive samples are indicated by the (*) and color-filled bars.

After an initial screening of serum samples by RT-PCR, serological analysis was performed on all 68 serum samples along with 15 control samples from healthy blood donors obtained from Morelia, as shown in Figure 2. We tested all the serum samples by using commercial CHIKV IgM and IgG ELISA Kits for the serodiagnosis of CHIKV infection, and the mean standard units (SU) were calculated (Figure 4). Interestingly, among seven CHIKV RT-PCR positive samples, four out of seven CHIKV-infected patients (sample numbers 8, 10, 16, and 19) were identified by IgG commercial kit, while only one sample (sample number 10) tested positive as CHIKV infection. Overall, 30/68 (44.1%) samples that were both RT-PCR positive and negative were shown to be positive (mean SU > 11) on the commercial IgG ELISA, with one sample in the grey zone (inconclusive) (Figure 4 Top). On the other hand, 7/68 (10.3%) of samples tested positive (mean SU > 11) and four samples were inconclusive on the commercial IgG ELISA kit (Figure 4, bottom). All 15 serum samples from healthy volunteers yielded negative results by ELISA (Supplementary Table S1).

### 3.3. CHIKV Serological Diagnosis Using the in-House ELISA

**Figure 5:**
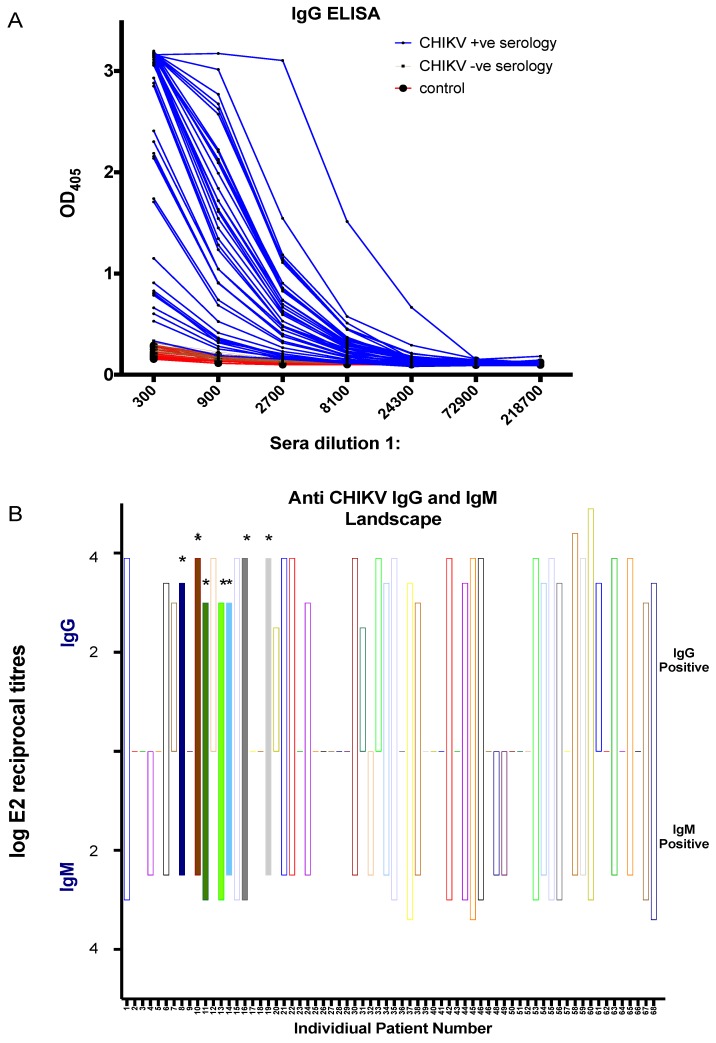
In-house recombinant CHIKV envelope protein 2 (E2)-based ELISA. (**A**) IgG ELISA assay to assess the reactivity of all the human serum samples in this study. Positive CHIKV serology (blue), negative CHIKV serology (brown), and negative control serum samples from healthy volunteers (red). (**B**) Log reciprocal E2 endpoint titres for IgG (positive *y*-axis) and IgM (negative *y*-axis) are represented for all 68 serum samples. RT-PCR positive samples are indicated by the (*) and color-filled bars.

Next, we tested all the serum samples by using our in-house CHIKV IgM and IgG ELISA for the serodiagnosis of CHIKV infection. The graph in Figure 5A shows the OD405 values over sera dilutions in range of 300 to 218,700 for IgG-based ELISA, which reliably detected and differentiated the sera from CHIKV-infected patients from control sera and CHIKV seronegative sera. Endpoint reciprocal titres (Figure 5B) were calculated which showed that 57.4% and 54.4% of serum samples were positive for in-house IgG and IgM ELISA, respectively (Figure 5B top and bottom). Importantly, all the RT-PCR positive serum samples (8, 10, 11, 13, 14, 16, and 19) were shown to be positive in our in-house IgM and IgG ELISA (Table S1). This demonstrates that our in-house recombinant E2-based IgM and IgG may be more sensitive than the used commercial kit.

### 3.4. Correlation of CHIKV Antibody Titres with the Commercial Kits

**Figure 6:**
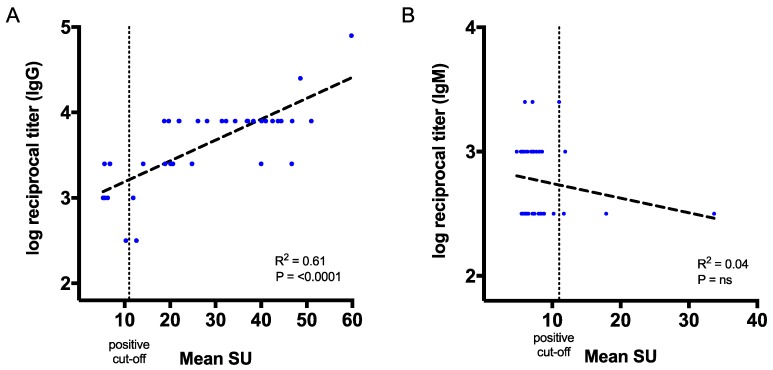
Correlation between the endpoint titres from in-house ELISA assay and mean standard unit (SU) from the commercial CHIKV kit. (**A**) Correlation between the in-house IgG endpoint titres and the commercial IgG mean SU. (**B**) Correlation between the IgM endpoint titres and the commercial IgM mean SU. The *p* values and R^2^ values reflect Pearson correlation tests. A positive cut-off is defined as >11 SU.

Next, we sought to correlate CHIKV antibody titres from our ELISA assay to the mean SU from the qualitative ELISA results from the commercial kits (Figure 6). Values of the reciprocal antibody titres and the mean SU for IgG showed a strong correlation (*p* < 0.0001 and R^2^ = 0.61) for the serologically positive patient samples that we tested (n = 39) (Figure 6A). On the other hand, no correlation was observed between our IgM ELISA and commercial IgM kit, which may be due to significant differences between the number of positive samples (7/68 versus 37/68) (Figure 6B). The results suggest that E2-specific antibodies could be measured by reciprocal endpoint titres in our in-house IgG ELISA assay, which correlates with the commercial IgG mean SU.

## 4. Discussion

In this study, we aimed to test the potential for the clinical use of an in-house ELISA assay with an E2 CHIKV protein and compare it with an available commercial ELISA kit using samples of a cohort from a region of Mexico that is endemic for CHIKV. We have recently reported a recombinant CHIKV E2 protein produced in a mammalian expression system based on HEK293 cells [26]. In this study, we tested whether our recombinant CHIKV E2 could be used to detect anti-CHIKV antibodies in clinical samples. Our results suggest that both IgG-based and IgM-based ELISA can reliably detect anti-CHIKV E2 antibodies in both acute and convalescent patient sera. Moreover, our assays provided higher sensitivity than the commercial kit used in this study, in particular for IgM ELISA. Many commercially available anti-CHIKV ELISA or immunofluorescence assays use whole virus-derived antigens, which can have high production costs due to virus culture and purifications steps, thus decreasing affordability and limiting their use in CHIKV-endemic regions in developing countries [18,27,28,29,30]. A previous study reported that the Abcam ELISA kit, in particular, showed batch-to-batch variability, with some kits demonstrating lower sensitivity than others, and the kit had to be re-released [18]. This could account for low sensitivity (one out of seven) in detecting IgM antibodies in RT-PCR positive samples by the Abcam IgM kit. Alternatively, this may be due to the low level of IgM antibodies in the acute CHIKV serum samples, which were insufficient to be detected by whole virus antigen used in the kit, as the actual amount of E2/E1 glycoproteins in the whole virus antigen plus the capsid and the same concentration of purified recombinant E2 may differ significantly. Previous studies have evaluated the *E. coli*-based recombinant E1 and E2 for Indirect ELISA [28,31]. It was shown that recombinant CHIKV E2 had higher sensitivity and specificity than CHIKV E1-based ELISA, and the results were comparable to the native antigen-based ELISA [28]. Another study showed that IgG E2 ELISA provided ~90% sensitivity and 100% specificity in concordance with CHIKV-specific neutralization assay, whilst IgM to E2 showed similar sensitivity to a commercial immunochromatography for CHIKV IgG and IgM detection [31]. Other studies used the baculovirus-derived CHIKV E1 for ELISA [32,33]. Taken together, these studies provide strong pieces of evidence that recombinant protein-based ELISA may be safe and cost-effective, along with a high degree of sensitivity and specificity for setting up CHIKV diagnostics. We have been using a mammalian-based cell culture system to purify large quantities of viral recombinant proteins that could be used in diagnostic assays [26,34,35]. Protein expression in a mammalian system ensures the post-translational modifications such as N-glycosylation [35]. It was previously shown that insect cell-derived CHIKV VLPs mainly consisted of oligomannose glycans, while HEK293-derived VLPs contain the mixture of oligomannose, hybrid, and complex glycans [36].

The in-house developed ELISA has higher sensitivity, and is also more cost-effective than using the commercial CHIKV assays. However, our E2-based recombinant ELISA assay would not detect anti-CHIKV E1 antibodies and some conformational epitopes targeting the E1/E2 heterodimers. We have recently reported the formation of virus-like articles by expressing the full structural cassette of CHIKV [26]; it will be interesting to test those as captured antigens in the ELISA assay to compare with our in-house E2-based ELISA and commercial ELISA kit to address the recognition of both anti-E1 and anti-E2 antibodies conformational epitopes. In order to validate our ELISA assay further, it will be useful to test negative controls such as serum samples from ZIKV or DENV-infected individuals. In addition, it will be very informative to determine PRNT titres for positive serology serum samples [37] that can support a sensitivity and specificity assessment of our in-house ELISA assay in comparison to PRNT as well as correlating antibody and PRNT titres. It will also be interesting to explore the cross-reactivity toward the antibodies against other alphaviruses, as they could be co-circulating in CHIKV endemic areas and determine the specificity of our ELISA assay [38,39]. We have detected ~50% seropositive using our in-house IgM and IgG ELISA for the febrile patients in Lázaro Cárdenas. As CHIKV co-circulates with other arboviruses in various endemic regions, there is often difficulty in differentiating between acute and previous CHIKV infections when their symptomatology is similar. Nonetheless, our ELISA assay could be helpful for the serodiagnosis of CHIKV in acute patients in the endemic regions of Mexico where there is no availability of specialized laboratories for RT-PCR, which may be important for effective patient management. In conclusion, our in-house CHIKV-specific ELISA can be a useful tool for the serodiagnosis of CHIKV infection, for seroprevalence studies in endemic regions to contribute to the management of the potential future epidemics, and for the monitoring of vaccine immunogenicity during CHIKV vaccine clinical trials.

## Supplementary Materials

The following are available online at https://www.mdpi.com/1999-4915/11/5/407/s1, Table S1: Comparative evaluation of recombinant E2 based in-house ELISA with respect to the commercial kit for all the serum samples used in this study.
