# Supplementary material for: Development of an E2 ELISA Methodology to Assess Chikungunya Seroprevalence in Patients from an Endemic Region of Mexico
Source: Viruses. 2019 May 1;11(5):407. doi: 10.3390/v11050407 (PMC6563309; doi:10.3390/v11050407)
Supplement: Supplementary file 1 [file viruses-11-00407-s001.pdf]

Supplementary Table S1. Comparative evaluation of recombinant E2 based in-house ELISA with respect to the commercial kit for all the serum samples used in this study. Standard units (SU): Negative: <9, Positive: >11, Grey zone (inconclusive): 9–11 for commercial kit and reciprocal endpoint antibody titres for in-house ELISA are shown.

Sample Number	Age Sex	Days after Onset of Symptoms	CHIKV RT-PCR (+/-)	IgM Commercial Kit (Mean SU)	IgG Commercial Kit (Mean SU)	IgM CHIKV E2 (Ab Titre)	IgG CHIKV E2 (Ab Titre)
1	2F	1	-	6.3	46.8	3.0	3.9
2	26F	3	-	4.7	6.3	-	-
3	26F	3	-	5.5	5.4	-	-
4	41F	1	-	17.9	5.6	2.5	-
5	35F	7	-	5.3	5.9	-	-
6	20F	4	-	6.2	6.8	2.5	3.4
7	28F	4	-	10.8	11.9	-	3.0
8	36F	5	CHIKV	5.9	20.1	2.5	3.4
9	29F	4	-	4.8	5.9	-	-
10	5M	4	CHIKV	11.7	51.0	2.5	3.9
11	15M	3	CHIKV	6.4	5.7	3.0	3.0
12	53F	4	-	10.7	21.9	-	3.9
13	25M	2	CHIKV	8.1	5.5	3.0	3.0
14	61F	3	CHIKV	5.5	5.5	2.5	3.0
15	39M	2	-	5.4	31.4	3.0	3.9
16	21F	5	CHIKV	4.8	40.1	3.0	3.9
17	19F	1	-	6.0	5.9	-	-
18	54F	4	-	6.1	5.5	-	-
19	26F	5	CHIKV	8.7	19.7	2.5	3.9
20	26F	2	-	7.8	12.6	-	2.5
21	49F	1	-	6.5	42.5	2.5	3.9
22	21M	2	-	8.0	43.7	2.5	3.9
23	41M	2	-	7.5	5.7	-	-
24	7M	2	-	10.2	5.3	2.5	3.0
25	35F	1	-	5.5	5.4	-	-
26	7M	4	-	5.0	6.0	-	-
27	41M	3	-	5.4	5.5	-	-
28	28F	5	-	4.7	5.5	-	-
29	30M	1	-	6.3	5.6	-	-
30	9F	2	-	11.7	37.1	2.5	3.9
31	89M	5	-	12.1	10.3	-	2.5
32	11M	3	-	33.7	6.0	2.5	-
33	50M	2	-	7.2	41.0	-	3.9
34	34F	4	-	7.4	40.0	2.5	3.4
35	8F	4	-	5.6	26.1	3.0	3.9
36	33M	2	-	4.3	5.3	-	-
37	7F	2	-	6.0	5.6	3.4	3.4
38	14M	3	-	5.5	5.6	2.5	3.0
39	68M	4	-	4.8	6.6	-	-
40	6M	2	-	4.5	5.3	-	-
41	35M	4	-	10.2	6.4	-	-
42	11F	3	-	7.3	36.9	3.0	3.9
43	15F	2	-	5.7	5.9	-	-
44	25M	5	-	7.1	14.1	3.0	3.4
45	12F	2	-	7.1	38.3	3.4	3.9
46	9M	5	-	5.9	44.4	3.0	3.9
47	30M	3	-	5.4	7.0	-	-
48	28M	5	-	7.4	5.6	2.5	-
49	41F	4	-	6.3	5.6	2.5	-
50	28M	2	-	8.5	5.4	-	-
51	43M	5	-	8.1	5.8	-	-
52	60F	5	-	6.7	6.0	-	-
53	23M	4	-	7.3	28.1	3.0	3.9

54	31M	3	-	6.1	20.6	2.5	3.4
55	31F	3	-	8.5	32.3	3.0	3.9
56	35F	3	-	11.9	18.9	3.0	3.4
57	28M	4	-	5.0	5.9	-	-
58	14F	1	-	7.1	48.6	2.5	4.4
59	22M	4	-	5.6	22.0	2.5	3.9
60	17F	2	-	7.7	59.8	3.0	4.9
61	48M	1	-	6.9	24.8	-	3.4
62	51M	9	-	6.5	5.8	-	-
63	67M	1	-	8.8	18.7	2.5	3.9
64	47M	1	-	5.1	5.4	-	-
65	53F	2	-	8.3	34.3	2.5	3.9
66	54M	2	-	6.4	8.1	-	-
67	25F	11	-	6.9	6.3	3.0	3.0
68	17M	5	-	11.0	46.7	3.4	3.4
HD-226		N/A	N/A	6.7	6.0	-	-
HD-236		N/A	N/A	5.5	6.1	-	-
HD-241		N/A	N/A	4.7	5.3	-	-
HD-254		N/A	N/A	6.3	5.4	-	-
HD-246		N/A	N/A	4.5	5.1	-	-
HD-234		N/A	N/A	5.7	5.2	-	-
HD-232		N/A	N/A	5.7	5.7	-	-
HD-258		N/A	N/A	5.8	5.4	-	-
HD-233		N/A	N/A	8.7	5.7	-	-
HD-239		N/A	N/A	5.4	5.5	-	-
HD-248		N/A	N/A	4.8	6.5	-	-
HD-247		N/A	N/A	4.8	5.7	-	-
HD-242		N/A	N/A	7.5	6.5	-	-
HD-260		N/A	N/A	5.5	5.9	-	-
HD-261		N/A	N/A	5.6	7.5	-	-
